# Applications of brain organoids in neurodevelopment and neurological diseases

**DOI:** 10.1186/s12929-021-00728-4

**Published:** 2021-04-22

**Authors:** Nan Sun, Xiangqi Meng, Yuxiang Liu, Dan Song, Chuanlu Jiang, Jinquan Cai

**Affiliations:** 1 Department of Neurosurgery, The Second Affiliated Hospital of Harbin Medical University, Harbin, 150086 China; 2 Department of Microbiology, Tumor and Cell Biology (MTC), Biomedicum, Karolinska Institutet, 171 65 Stockholm, Sweden

**Keywords:** Brain organoid, Pluripotent stem cell, Brain development, Neurological disorders, Glioblastoma

## Abstract

A brain organoid is a self-organizing three-dimensional tissue derived from human embryonic stem cells or pluripotent stem cells and is able to simulate the architecture and functionality of the human brain. Brain organoid generation methods are abundant and continue to improve, and now, an in vivo vascularized brain organoid has been encouragingly reported. The combination of brain organoids with immune-staining and single-cell sequencing technology facilitates our understanding of brain organoids, including the structural organization and the diversity of cell types. Recent publications have reported that brain organoids can mimic the dynamic spatiotemporal process of early brain development, model various human brain disorders, and serve as an effective preclinical platform to test and guide personalized treatment. In this review, we introduce the current state of brain organoid differentiation strategies, summarize current progress and applications in the medical domain, and discuss the challenges and prospects of this promising technology.

## Background

Determining the mystery of human brain development and neurological disorders has consistently been a fascinating challenge in medical research for hundreds of years. The scientific communities have been making enormous efforts on this issue, and various in vivo and in vitro models, such as cellular and animal models, have been established for medical research and have substantially improved our understanding of the physiological and pathological processes in the human brain [1, 2]. Nevertheless, some preclinical findings acquired from those models failed to be translated into clinical practice successfully, partially due to differences in the subtle structure and cellular composition across species existing between the brains of humans and those models [3]. Additionally, although the human brain is an ideal subject for studying neuropathology, the relative inaccessibility for research purposes and the difficulties in cultivation and manipulation limit its application.

Therefore, a new model that can better recapitulate the characteristics of the human brain is urgently needed. In recent decades, the tremendous potential of human PSCs to self-renew indefinitely and to differentiate in multiple directions has attracted great attention in the field of biological research and medical applications [4, 5]. Publications have reported the availability of human PSCs in studying molecular mechanisms and therapeutic approaches for neurodegenerative diseases such as Alzheimer’s disease and the practice of human PSCs in regenerative medicine for the traumatic injuries of the central nervous system [6, 7]. Furthermore, stem cell technology has progressed and focuses on the complete set of cell types of organs rather than merely pure populations of cell types, and a new remarkable model named brain organoids derived from human PSCs has been constructed and proven to be promising for biological and medical research.

In this review, we summarize the existing generation methods, tissue structure, and functional neuronal activities of brain organoids. In addition, we introduce the current state of brain organoid applications in exploring human brain development, modeling neurological disorders, and drug screening. Additionally, we also discuss the challenges and prospects of this promising technology in the future. This review is beneficial for our understanding and utilization of this model.

## Definition and history of organoid technology

An organoid is a self-organized 3D tissue with a collection of stem and organ-specific cell types derived from stem cells or organ progenitors to simulate the architecture and functionality of the native organ to some extent. Cell sorting and spatially restricted lineage commitment have proven to be foundational processes of organoid self-organization. Three features characterize an organoid: containing multiple organ-specific cell types, recapitulating some specific function, and spatial organization similar to a human organ [8]. As a novel in vitro model, organoids hold multiple advantages over traditional two-dimensional (2D) cell cultures and animal models. Compared with 2D cell cultures, organoids provide physiologic conditions closer to the human organism and support cell–cell and cell–matrix interactions. Although both animal models and organoids are available for manipulation with genome editing technology and are equipped with the capability to provide a physiologic environment, organoids can be extensively expanded in culture and maintain genomic stability, making them suitable for high-throughput screening and building biobank. More importantly, there are differences in the structure between human and mouse brains; for example, (1) the inner fiber layer (IFL) and outer subventricular zone (OSVZ) in the human brain are absent in the mouse brain [9]; (2) the human cortex is expanded relative to the mouse with a > 1000-fold increase in the area and number of neurons [10]; and (3) some cell types, such as interlaminar astrocytes [11], and rosehip neurons [12], have specialized features in humans compared to mice. These differences are some of the reasons for the failures with the use of mice in preclinical studies of effective drug screening and the misunderstanding of human neurological disorders [10].

In 1907, Wilson demonstrated the potential of dissociated sponge cells to self-organize to regenerate a whole organism, which is the earliest observation of the phenomenon of reassembly of cleaved tissue [13]. In 1981, mouse pluripotent stem cells were isolated from early mouse embryos, and then human embryonic stem cells were isolated from human blastocysts in 1998, which laid the foundation for the emergence and development of organoid technology [14, 15]. A groundbreaking discovery in the organoid field occurred in 2009, Clevers et al. generated gut organoids from adult intestinal stem cells upon 3D culture in Matrigel, which was the first time that the organoid had actually been constructed in history [16]. Since then, the field of organoids has been developing rapidly, and more other organoids have been generated and cultured, including the lung [17], kidney [18], prostate [19], and brain [9].

## Generation of the brain organoid

**Fig. 1:**
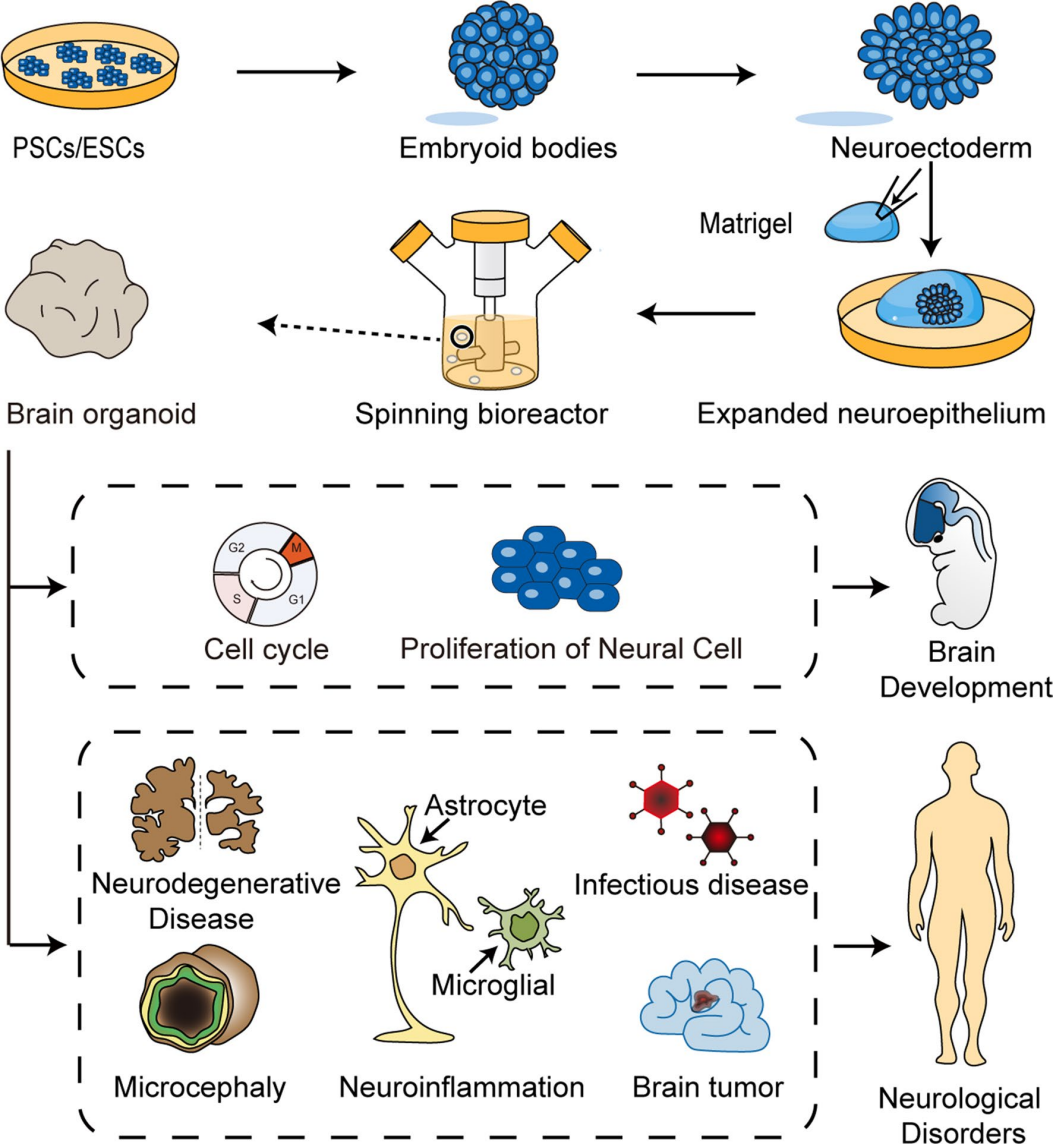
Schematic of the generation and applications of brain organoids. ESCs from human embryonic tissues and PSCs from adult tissues first divide and aggregate into EBs, are placed in neural induction media to induce neuroectoderm formation, and are subsequently transferred into Matrigel droplets to expand the neuroepithelium. The cultures during this period are early brain organoids. Last, these tissues will be cultured in the spinning bioreactor to enhance nutrient absorption for further maturation and preservation. Brian organoids can be used to recapitulate the process of human brain development and to investigate the factors affecting neurogenesis. Brain organoid technology can be exploited to model a variety of human neurological disorders, such as tumors and microcephaly, to explore the pathogenic mechanism and identify an effective treatment for patients

Based on stem cell technology and the principles of cell self-organization, brain organoids, in vitro 3D culture systems resembling human brains, have been generated and sequentially improved. The earliest undertaking in achieving the generation of a brain organoid called a cerebral organoid was reported by Lancaster et al. in 2013. The method began with the generation of embryoid bodies (EBs) from PSCs or ESCs. Every EB contains three germ layers, including the endoderm, mesoderm, and ectoderm. Since that neural tissue develops from the ectoderm in the human body, EBs were placed in neural induction media to induce neuroectoderm formation [20]. Then, the differentiated EBs were embedded in droplets of Matrigel, which provided a scaffold support for the growth of complex organoid structures. After that, cultured tissues began to form clearly expanded neuroepithelial buds containing fluid-filled cavities reminiscent of brain ventricles. Finally, the embedded organoids were transferred into a spinning bioreactor to enhance oxygen and nutrient absorption for further maturation and preservation (Fig. 1). Cerebral organoids began to exhibit neuronal differentiation after 1 month of culture. Over the next 1 to 2 months, the cerebral tissue gradually expanded and thickened to form different brain regions, including the forebrain, choroid plexus, hippocampus, ventral forebrain, and retina. The organized apical progenitor zone was surrounded by basally located neurons in a large continuous cortical tissue within an organoid, as evidenced by immunofluorescence staining for neurons (TUJ1) and progenitors (SOX2) [21]. Outer radial glial cells and inner radial glial cells are located in and undergo mitosis while residing outside and inside the ventricular zone, respectively. The growth of cerebral organoid tissues generated with this method stopped by 2 months and steadily diminished in size after 5 or 6 months, but these organoids could be maintained for up to 1 year in the spinning bioreactor [21].

Recently, the development of 3D culture systems has led to the production of brain organoids similar to different regions of the human brain [22–24]. Jo et al. reported a method for the generation of human midbrain-like organoids (hMLOs) by culturing hPSCs in media with the addition of sonic hedgehog (SHH) and FGF8 (two factors that have been confirmed to promote stem cell differentiation toward a mesencephalic fate identity) [24]. Approximately 80% of the cells in 35-day-old hMLOs proved OTX2+ (marker of the midbrain) and 35% within neuroepithelia were double positive for EdU (marker of cell proliferation) and OTX2, revealing the identity of midbrain progenitors located in these organoids. The midbrain dopaminergic neuroprogenitor marker FOXA2 was detected in 4-day-old hMLOs. Interestingly, the expression of FOXA2 was restricted to specific regions of neuroepithelia on the 14th day of cultivation, and these FOXA2-expressing cells began to migrate to the mantle zone (MZ), where tyrosine hydroxylase, the rate limiting enzyme for dopamine synthesis and a marker of mature midbrain dopaminergic neurons, was expressed. These facts demonstrated that hMLOs reproduced the time-dependent differentiation of dopaminergic neurons in the midbrain, similar to the process of early development in human midbrains. In addition to the midbrain, the addition of WNT3A, SHH, and purmorphamine to the culture media contributed to the differentiation of iPSCs to the hypothalamic lineage, generating hypothalamic organoids [23]. The markers that are expressed during early human hypothalamus development, such as, RAX1, SOX2, and NESTIN, were positive in most cells of 8-day-old hypothalamic organoids. The cerebellar organoid was also generated by culturing human ESCs with sequential addition of growth factors in Muguruma et al.’s work [9].

Some researchers have tried to combine these independent brain regional organoids to explore the mechanism and complex processes of brain development and neurological disorders [25–27]. Bagley et al. cultured ventral and dorsal embryoid bodies (EBs) together within a single Matrigel droplet, generating a dorsal–ventral axis in the fusing ventral and dorsal cerebral forebrain organoid [25]. Cells positive for γ-aminobutyric acid (GABA)ergic markers (GAD1/VGAT) migrate from the ventral to the dorsal region in fused forebrain organoids and exhibit a ventral forebrain-derived interneuron identity supported by the lack of RELN expression, recapitulating the process of interneuron migration from the ventral to dorsal regions in the human forebrain [25]. Similarly, Xiang et al. cocultured medial ganglionic eminence (MGE) and cortical organoids, generating fused MGE-cortical organoids (hfMCOs) [26]. Single-cell RNA sequencing and ATAC-seq revealed the great capacity of these hfMCOs to recapitulate fetal brain organization and transcriptomes. The process of human interneuron migration between MGE and cortical neurons can be reproduced in these fused organoids, providing deeper insight into the molecular dynamics in the development of the human brain [26]. Xiang et al. modeled axon projections between the human thalamus and cortex in fused thalamic and cortical organoids [27]. Collectively, brain regional organoids can be harnessed in recapitulating the structure and function interaction between specific areas of human brains, exhibiting great potential in revolutionizing the study of human brain development, neural function pathway exploration, and central neurological disorders.

However, the cost and the required volume of incubator space of the Lancaster et al. spinning bioreactor are relatively high, which limits its widespread use. Qian et al. developed a miniaturized spinning bioreactor named Spin Ω, which matched the standard of a 12-well tissue culture plate and achieved a reduction in culture solution consumption. Furthermore, they treated human iPSCs with region-specific patterning factors in the medium and managed to generate brain-region-specific organoids, including the forebrain, midbrain, and hypothalamic organoids [23]. Nevertheless, a visible defect of these models is the lack of vascularization, restricting the delivery of sufficient oxygen and nutrition to the internal organoids. In terms of this issue, Mansour et al. transplanted human brain organoids onto the cortex of the adult mouse brain and induced impressive outgrowth of blood vessels into the organoid tissue. Vascularized brain organoids exhibit better neuronal survival and maturation [28].

In addition, Cakir et al. managed to generate a complex vascular-like network in human brain organoids [29]. It has been reported that the expression of ETS variant 2 (ETV2) can directly reprogram human postnatal cells to functional, mature ECs after an intervening transgene-free period [30]. Cakir et al. demonstrated that the overexpression of ETV2 can induce the transformation from human dermal fibroblasts to endothelial cells [29]. Therefore, they generated a special organoid comprising 20% human ETS variant 2 (ETV2)-infected hESCs and 80% untreated hESCs. Then, the infected cells were induced to express ETV2 on the 18th day of culture, and these ETV2-expressing hESCs in cortex organoids differentiated into endothelial cells and then contributed to forming a functional vascular-like network [29]. Functional vessels are beneficial to the growth of organoids, accelerate the maturation of cortical neurons and promote the emergence of several blood–brain barrier characteristics, including an increase in the expression of tight junctions and transendothelial electrical resistance [29]. Therefore, vascularized brain organoids reflect the physiology of the human brain more accurately and present a robust model to study advanced brain functions in vitro. It is fascinating and worthwhile to investigate the developmental duration and maximum maturity of vascularized brain organoids in the future.

## The composition of brain organoid

Currently, increasing emerging technologies such as single-cell sequencing have been widely applied in medical research [31]. Taking advantage of them to study brain organoids will help us to explore these models more deeply and achieve a better understanding of their practicality [32]. After 1 month of culture, cerebral tissues in organoids expanded and showed early brain regionalization, including the forebrain, choroid plexus, hippocampus and retinal zones [21]. Quadrato et al. probed the composition of the cell and tissue structure of typical brain organoids generated by the modified protocol published by Lancaster et al. [33]. Over 80,000 individual cells isolated from 31 human brain organoids at 3 and 6 months were molecularly profiled with droplet-based single-cell mRNA sequencing (Drop-seq). Clustering all cells from the 6-month organoids identified six main transcriptionally distinct populations belonging to the neuroectodermal lineage, such as astrocytes, progenitors, and neural retinas, which resembles the appropriate endogenous counterparts from the human fetal brain. Intriguingly, although some cell types can be found in both 6- and 3-month organoids, some presented only at 6 months including callosal projection neurons, Müller glial, and bipolar cells. Similarly, photoreceptor markers (CRX) and mature astrocyte markers (GFAP, AQP4, and AGT) were present only at 6 months. These results revealed that long-term culture enables the continuous development of brain organoids to expand cellular diversity and to promote neuronal maturation [33]. In addition, synapses and dendritic spines, structural traits of mature neurons, were also found in organoids at 8 months, suggesting that the mature brain organoid can be used to investigate advanced nervous function. More interestingly, neuronal activity within organoids could be controlled by stimulating photosensitive cells with light, indicating that a brain organoid may be a valid object to probe the functionality of individual neuronal circuits [33].

The doubt regarding whether brain organoids can achieve a degree of reproducibility in cell composition between different organoid cultures that is similar to the processes in the human embryo has always been of interest to scientific researchers [34]. Recently, Silvia Velasco et al. quantify cellular composition across individual organoids, promoting our understanding of the reproducibility of different brain organoid models [35]. They generated nine individual organoids by culturing HUES66 and PGP1, two separated stem cell lines, in the same spinner-flask bioreactors over a duration of 3 months. The organoids derived from HUES66 stem cells were developed in one batch, and the organoids from PGP1 stem cells were divided into two independent batches in time. They performed high-throughput scRNA-seq analysis on a total of 78,379 cells from nine individual organoids (three organoids from PGP1 of batch 1; three organoids from PGP1 of batch 2; and three organoids from HUES66) and defined 11 main transcriptionally distinct cell types. Cocluster analysis of transcriptional signatures for cell types revealed that the cell composition of these nine brain organoids is highly reproduced across different stem cell sources and generation batches. In addition, immunohistochemistry results showed equal high consistent expression of cell type-specific markers, including MAP2 (neuronal), EMX1 (dorsal forebrain progenitor), SOX2 (radial glia), and Ki67 (proliferation) [35]. These facts indicated that human brain organoids could not only mimic the diverse cell types of developing human brains but also show good consistency in reproducing cell composition across individual organoids and experiments. Furthermore, Giorgia Quadrato et al. quantified the degree of variability between brain organoids across cultures from different bioreactors [33]. Most of the 6-month-old cerebral organoids cultured in the No.4 bioreactor were less differentiated and contained a large number of progenitor cells, but the same-aged and same-iPSC-derived organoids cultured in the other three bioreactors had much higher proportions of differentiated cells. Ten transcriptionally distinct populations were defined by clustering all cells from 6-month organoids with single-cell mRNA sequencing profiles. Most cell clusters were consistently present in all organoids developed in four different bioreactors, but one cell cluster (including radial glial cells, interneurons, intermediate progenitors, callosal neurons and corticofugal neurons) was only significantly enriched in the organoids developed in the NO. 3 bioreactor and exhibited low levels in organoids generated in the other three bioreactors [33]. These results suggest that the organoid growth environment plays a key role in the variability of cell composition among different organoid cultures. The availability of brain organoids has opened up an avenue in modeling a variety of neural disorders and provided a wealth of opportunities for screening effective drugs. For example, several different kinds of GBM models derived from different kinds of brain organoids show great results in screening antitumor drugs [36, 37]. However, in view of the organoid-to-organoid variability, the results of testing drug efficacy with diverse organoids may be different. Therefore, to ensure that the drugs screened by organoids can be transformed into effective clinical applications, it is necessary to further explore the influence of the variability between organoids on drug efficacy [38].

## Applications of brain organoids

Brain organoids have been widely utilized across various research disciplines and medical applications in the past few years, relying on the capacity to resemble physiological tissue organization and to simulate brain function to some extent. These range from basic development research to personalized medicine, with research and data being widely presented. Here, we summarize the classic and latest brain organoid applications that refer to brain development, disorders, and drug screening.

### Exploring human brain development

Brain organoids have opened up a new avenue for investigating early human brain development. Tumor protein p53 (TP53), a well-known cancer suppressor gene, has been studied in carcinogenesis and cancer development, but its effect on human brain development is less understood. Recently, Navarro et al. explored the role of TP53 in human brain development with organoids [39]. Control short hairpin RNA (shCtrl) and short hairpin RNA against TP53 (shTP53) were transduced into human iPSCs, and then they were used to generate controls and TP53-knockdown (TP53KD) brain organoids, respectively. After 30 days of culture, the immunohistochemistry results showed that the neural stem cell (NSC) layer was disorganized, and SOX2 + NSCs were distributed inside and outside the tubular region of TP53KD organoids. In addition, the TBR1+ postmitotic neurons and TBR2+ intermediate progenitors were reduced significantly in TP53KD organoids compared with controls. To probe whether the reduction in both neurons and progenitors was due to changes in the number of NSCs, Navarro et al. detected the proliferation and apoptosis of NSCs from TP53KD organoids and controls, and no significant difference was found between them. Next, they investigated whether TP53 affected the cell cycle distribution of cells in organoids. Indeed, an accumulation of cells in G1 phase and a reduction of cells in S phase were found in TP53KD organoids, revealing the negative effect of TP53KD on cell cycle and showing the vital function of TP53 in regulating proper human brain development.

Previous studies have reported that most G protein-coupled receptors (GPCRs) can show activity even without ligand binding. This characteristic was termed receptor constitutive activity. Dopamine D1 receptor (DRD1), a typical GPCR, is copiously expressed in the human central nervous system [40]. Qinying Wang et al. probed the effect of dopamine D1 receptor (DRD1), a typical GPCR that is abundantly expressed in the human central nervous system, on the human brain development process with brain organoids [41]. Given that the proliferation of NSCs affects the morphology of human brain organoids [42], Qinying Wang et al. inhibited the constitutive receptor activity of DRD1 by treatment with inverse agonists or knockdown of the DRD1 gene in the organoid and found that DRD1 induced a significant expansion and folding morphology appearance of the organoid [41]. Furthermore, the treatment of organoids with PKC inhibitors lead the same consequences as above, demonstrating that the PKC-CBP pathway was involved in the regulation of normal brain development by DRD1 [41]. Taking advantage of brain organoids, a more detailed and abundant neural development process can be observed in vitro, including dynamic changes and distribution of nerve cells as well as the organization and morphology of brains. From this perspective, many genes or molecules whose functions have been studied in cell or animal experiments can be investigated deeply with brain organoids, which is a promising direction.

### Modeling glioblastoma

Glioblastoma (GBM), the highest grade glioma (grade IV), is one of the most frequent malignant primary tumors in the central nervous system in adults [43]. Despite surgical resection followed by chemotherapy and radiotherapy, a patient’s median survival remains no more than 15 months after initial diagnosis [44]. Several preclinical model systems for GBM research have been developed, including cancer cell lines [45], patient-derived xenografts (PDXs) [46, 47], and genetically engineered mouse models (GEMMs) [48]. Although these models have provided crucial insights into our understanding of the biological mechanisms underlying GBM pathogenesis, they fall short on account of their lengthy process, high costs, and lack of a physical environment similar to the human body (Table 1). Fortunately, the emergence of GBM organoid models provides researchers with an alternative tool to understand this aggressive brain tumor.

**Table 1:** Comparison of preclinical glioblastoma models

	Cancer cells	GEMM	Tumor organoid	PDX
Physiologic representation	No	Great	Good	Great
Immune environment	No	Yes	No	No
Tumor heterogeneity	No	Bad	NA	Great
Oncogenesis time	NA	Long	Medium	NA
Tumorgenesis	No	Great	Great	No
Manipulability	Great	Limited	Good	Limited
Genome editing	Yes	Yes	Yes	No
Biobanking	Yes	No	Yes	No
High-throughput drug screening	Yes	Yes	Yes	No
Additional variables introduction	No	Yes	No	Yes

*NA* not available, *GEMM* genetically engineered mouse model, *PDX* patient-derived xenograft model

Genome-editing technology, such as the CRISPR/Cas9 and Sleeping Beauty transposon system, has emerged as a powerful and reliable tool to elucidate gene function and to discover mechanisms of human diseases. The combination of organoids and genome-editing techniques could promote human cancer research. Ogawa et al. overexpressed the Ras oncogene and simultaneously disrupted the TP53 tumor suppressor with CRISPR/Cas9 technology, successfully generating a genetically defined model of human GBM in 4-month-old cerebral organoids [49]. By analyzing the expression profiles of the tumor cells, organoid-derived tumors were proven to fall within the category of mesenchymal subtype GBMs. Sixteen weeks after model construction, the cerebral organoid was composed of 86.8% tumor cells and showed marked buds that were never observed in healthy cerebral organoids, which was reminiscent of invasive edges in human GBM. Furthermore, patient-derived and organoid-derived tumor cells can spontaneously attach to and spread through intact organoids, which mimics the invasive behavior of human GBM and suggests the possibility of testing the properties of human primary tumor explants in brain organoids. This technology recapitulated the putative initiating genetic events and the natural history of tumor development of human GBM, processes that are ordinarily invisible in human patients. Additionally, Shan Bian et al. combined Sleeping Beauty transposon-mediated gene insertion and CRISPR/Cas9-mediated gene mutagenesis technology to construct tumorigenic genes of brain tumors, including 18 single-gene mutations or amplifications, as well as 15 of the most common clinically relevant combinations observed in brain tumors, and then introduced them into EBs to generate neoplastic cerebral organoids [50]. Interestingly, only four mutations worked in brain tumorigenesis after 1 month of culture, and three quarters of the mutation types contributed to GBM, which showed the capacity of cerebral organoids as a platform to test the tumorigenic capacity of different gene aberrations. The cell composition of organoid-derived tumors initiated with defined gene aberrations is homogeneous, which makes organoids suitable for studying the effects of specific genes on tumors. However, human GBM exhibits high inter- and intratumoral heterogeneity, and a model constructed using gene-editing technology cannot recapitulate it well [51, 52].

GBM models constructed by combining patient tumor tissue with brain organoids can compensate for this. Silva et al. cocultured human GBM spheroids and mouse embryonic stem cell-derived early-stage cerebral organoids and managed to model the process of GBM infiltration and invasion [53]. For the last few years, there has been much evidence that patient-derived glioma stem cells (GSCs) can well represent the phenotypic and physiological characteristics of parental tumors [54]. Characterized by the capacity for self-renewal and differentiation into multilineages, GSCs contribute to tumorigenesis, maintenance, and infiltration in vivo. It has been reported that the activity of GSCs is not completely autonomous in vivo, but rather considerably influenced by the interaction with host cells as well as the support of the three-dimensional extracellular matrix environment [55–57]. That, however, is what brain organoids can do. Based on this, Linkous et al. established a ‘‘GLICO’’ (cerebral organoid glioma) model by coculturing patient-derived GSCs with human embryonic stem cell (hESC)-derived cerebral organoids [36]. GFP-labeled GSCs were cocultured with individual, fully formed cerebral organoids for 24 h, and tumor-infiltrated organoids were monitored daily by immunofluorescence microscopy for evidence of tumor formation. The tumor take rate was 100% for GSC lines, and considerable tumor growth was detected 1 week after coculture. Subsequent neuropathological evaluation of tumor-bearing organoids revealed a hypercellular bulk tumor with an infiltrating edge of GSCs that invaded the normal tissue, thus recapitulating the tumor morphology observed in human patients with GBM [36]. In addition, tumor cells in GLICO derived from GSCs of different GBM patients exhibited different patterns of infiltration in the cerebral organoids. However, they highly copy the phenotypes of parental tumor samples, demonstrating that GLICO could recapitulate the patient-specific tumor invasive phenotype. In addition, GLICOs can preserve key patient-specific genetic and signaling components, such as EGFR amplification and phospho-RTK signaling. The gap junction mediated interconnecting tumor microtube network is reported to be a structure of in situ GBMs that facilitates communication between tumor cells and is beneficial to their proliferation and invasion [58]. A similar microtube network was observed in GLICO, and these microtubes penetrated deeply into healthy cerebral organoid tissues and provided multicellular connections among various tumor cells. In addition, effective travel of a calcium signal was detected in these tumor microtubes with time-lapse imaging. Patient derived GLICO showed the biological behaviors and histopathological features of GBMs in a manner that closely phenocopies surgical and autopsy specimens, attesting to the model’s clinical relevance and patient specificity. However, an obvious drawback of this model is the lack of a vascular system, limiting the growth and application of GLICO. Recently, the vascularized brain organoids have been established [29]. Hence, the problem might be solved by coculturing the vascularized brain organoids with GSCs. In addition, Gladiola et al. developed three different methods that allow assaying of GSC invasion behavior in brain organoids, including simultaneous coculturing of GSCs during brain organoid differentiation, supplementing GSCs as dispersed cells into brain organoids, and fusing GSC spheres to brain organoids. Their work provided controlled and uncomplicated protocols to characterize GSC invasions and demonstrated the reliability of brain organoids in modeling GBM [59].

Interestingly, Jacob et al. reported a novel method to generate patient-derived GBMorganoids (GBOs), which differs from the conventional building method of the GBM organoid model based on the brain organoids that were constructed as mentioned above [37]. Fresh surgically resected GBM tissues without mechanical or enzymatic dissociation into single cells were cut into approximately 1-mm-diameter pieces and then cultured in optimized medium (serum-free, no exogenous EGF/bFGF, and no extracellular matrix) on an orbital shaker. GBOs were generated within 1–2 weeks. Though this model is not “traditional”, which has several highlights. First, GBM tissues were not dissociated into single cells, maintaining the local tumor cytoarchitecture and the interaction between tumor cells. Second, GBOs can partially preserve the microvasculature and immune cell populations of parental tumor tissue, shedding light on a better understanding of the tumor microenvironment. Nevertheless, on account of the limited lifespan of resident immune cells, a decreased abundance of macrophage/microglia populations and lower expression of immune-related genes were detected in GBOs over time. Third, GBOs largely recapitulate the molecular features of their parental tumors, including inter- and intratumoral genomic and transcriptomic heterogeneity, representing a promising strategy for studying GBM pathogenesis and developing personalized therapy. Furthermore, an organoid biobank with 70 GBOs derived from different patients was established, which included abundant biological information of GBM, including histology, RNA-seq, and whole-exome sequencing, and will be a valuable resource for medical studies in the future [37].

### Modeling human neurodegenerative diseases

Neurodegenerative diseases (NDDs) encompass a group of conditions that are pathologically and clinically diverse, including Parkinson’s disease (PD) [60], Alzheimer’s disease (AD) [61], amyotrophic lateral sclerosis (ALS) [62] and other neurological disorders [63] characterized by the accumulation of misfolded proteins and the loss of functional neurons in the affected regions of human brains [64]. NDD is a common and growing cause of mortality and morbidity worldwide, particularly in the elderly [65]. To improve upon the current situation in which there are few therapies for NDDs and the treatment effect is not significant, the mechanisms underlying neurodegeneration and an effective drug-screening system are required for NDD treatment. Although traditional 2D cell culture and animal model systems have provided valuable insights into the main pathophysiological pathways related to these diseases, they have not been well translated into clinical applications [66, 67]. Fortunately, 3D brain organoids provide revolutionary tools for the study of human NDDs, allowing noninvasive analysis of patient-derived human tissues [68–70].

Alzheimer’s disease (AD) is a common NDD that causes dementia, which is characterized by abnormally folded amyloid-β (Aβ) peptide deposition and intracellular neurofibrillary tangles, caused by the aberrant processing and polymerization of normally soluble proteins [71]. AD currently affects more than 40 million people worldwide, and patients suffer from varying degrees of cognitive decline and severe memory impairment [72]. Researchers have been committed to understanding the pathological process of Alzheimer’s disease and developing effective drugs [73, 74]. Mutations in the amyloid-β precursor protein (APP) [75] and presenilin (PS) 1 genes [76] have been found to contribute to familial AD (FAD). Hoon Choi et al. reported an AD brain organoid derived from human neural stem cells that overexpressed human familial AD mutations in the APP and PS1 [77]. These familial AD organoids exhibited distinct deposition of Aβ and hyperphosphorylated tau protein that was characteristic of human AD brain tissues. In addition, treatment with β- or γ-secretase inhibitors could attenuate tauopathy and decrease the level of Aβ in organoids. This unique 3D brain organoid successfully recapitulated the key features of AD pathology, and was proven to be an available and effective in vitro model to promote the process of NDD research [77]. Furthermore, Cesar Gonzalez et al. reported the generation of an AD brain organoid produced directly from iPSCs derived from patients who developed familial AD, and pathological abnormal features including amyloid plaques and neurofibrillary tangles, were also detected in these organoids [78]. This research demonstrated the feasibility of developing patient-specific in vitro AD models with patient somatic cells, and provided a new platform for the discovery of target drugs and effective therapeutic intervention. Furthermore, Swagata Ghatak et al. found that the increased excitatory bursting activity was connected with the decrease in neurite length, which provided mechanistic insight into the hyperexcitability during the initial stages of AD [79].

Parkinson’s disease (PD) is the second most common NDD after AD, with a prevalence of approximately 2 per 1000 individuals worldwide [80, 81]. PD is characterized by the loss of dopamine in the substantia nigra and the dysregulation of fine motor control localized in the basal ganglia, which leads to the clinical parkinsonian symptoms, including bradykinesia, muscular rigidity, and resting tremors [82]. The LRRK2 G2019S gene mutation was reported to be associated with the progressive loss of dopamine neurons in the PD pathological process [73]. Recently, Kim et al. generated 3D midbrain organoids derived from iPSCs with the LRRK2 G2019S mutation to gain a deeper understanding of the role of the LRRK2 mutation in the pathogenic mechanisms of PD [69]. Compared with the control group (midbrain organoids without the LRRK2 G2019S mutation), the expression of dopaminergic neuron markers (TH, AADC, VMAT2 and DAT) in organoids with the LRRK2 G2019S mutation was significantly reduced on the 60th day. PD-like pathological features can also be detected in these organoids, including the abnormal location of pS129 a-synuclein vesicles and mitophagy with autophagy markers. In addition, after treatment with an LRRK2 kinase inhibitor, the accumulation of phosphorylated a-synuclein and the death of dopaminergic neurons were alleviated, indicating that brain organoids are a promising platform for drug screening [69].

Amyotrophic lateral sclerosis (ALS) also remains a common progressive neurodegenerative disease characterized by the loss of motor neurons and muscle atrophy [83]. Familial ALS accounts for approximately 10% of cases and is related to specific genetic mutations including TAR DNA-binding protein 43 (TDP-43) [84], and TANK-binding kinase 1 (TBK1) [85]. Tatsuya Osaki et al. developed neuromuscular junctions in a fusion 3D organoid model using human iPSC-derived muscle bundles and human motor neuron spheroids derived from sporadic ALS patients [70]. Compared with the control group, the expression of ISL1, choline acetyltransferase (ChAT) and synaptophysin I in motor neurons in organoids decreased significantly, revealing that increased motor neuron degradation and apoptosis, weakness of synaptic function, and impaired motor features were reproduced in these organoids. Moreover, muscle contractions were increased and neuronal survival was improved after treating these ALS organoids with the potential target drug candidates, bosutinib and masitinib, indicating that the treatment not only improved motor neuron neuroprotection but also suppressed miscommunication between neurons and muscles in fused organoids [70].

### Modeling microcephaly and neuroinflammation

Autosomal recessive primary microcephaly (MCPH) is a neurodevelopmental disorder characterized by a markedly reduced size of the cerebral cortex, but with normal architecture [86, 87]. Several gene mutations, such as CDK5RAP2, have been identified in MCPH patients, and most of which encode centrosomal proteins [88, 89]. Primary microcephaly mouse models with MCPH-related gene mutations have revealed the role of these genes in impacting the proliferation of neural progenitors but failed to recapitulate a severe reduction in brain size, as observed in human patients [90, 91]. Lancaster et al. modeled human microcephaly and managed to explain the disease phenotype partly with cerebral organoids [9]. They reprogrammed fibroblasts with heterozygous truncating mutations in CDK5RAP2 obtained from a microcephaly patient to be human iPSCs as the source of a patient-derived microcephaly organoid model [88, 89]. Immunohistochemical staining results showed that patient-derived microcephaly organoids displayed only occasional neuroepithelial regions. Additionally, organoid tissues exhibited decreased radial glial stem cells (RGs), and increased neurons at an earlier stage (22 days) of culture, indicating premature neural differentiation. In addition, the exclusively horizontal orientation, which is necessary for the early symmetric expansion of NSCs, was disrupted within patient organoids [92]. This research recapitulates the small-size brain phenotype and reveals the critical role of CDK5RAP2 in MCPH pathogenesis with brain organoids.

Some publications reported that Zika virus (ZIKV) infection was connected with a significant increase in newborns suffering from microcephaly and neurological diseases [93]. Previous studies have shown that Zika virus can induces stress response in organoids and human neurons [94, 95], and mechanistic studies have correlated microcephaly and cerebral cortex development deficits to increased unfolded protein response [96–98], a proteostasis failure rescue pathway. Tang et al. indicated that ZIKV infected human embryonic cortical neural progenitor cells (NPCs) in a 2D culture, leading to cell death and dysregulation of the cell cycle [99]. Moreover, several research groups infected immature cerebral organoids with ZIKV and found an overall decrease in organoid size, including ventricular and cortical plate thickness [100, 101]. These results observed in organoids are consistent with the clinical finding from the ZIKV-infected human fetal brain [102]. Intriguingly, Dang et al. found that Toll-like-receptor 3 (TLR3), which has been associated with neurodegeneration and neuroinflammation, was upregulated in organoids after ZIKV infection and that severe cell apoptosis and size shrinkage in ZIKV-treated organoids were relieved after treatment with a TLR3 competitive inhibitor [103, 104]. Furthermore, transcriptomic analysis showed that a few genes regulated by TLR3, including NTN1 and EPHB2, were related to neurogenesis and apoptosis in developing organoids. Brain organoids helped us recover the pathogenesis of microcephaly in which ZIKV disturbed neurodevelopment by perturbing a TLR3-regulated network.

Methamphetamine (METH) is a potent stimulant that induces a temporary euphoric state but also commonly leads to central nervous system disorders, such as psychosis, mental damage, and neurodevelopmental deficits [105]. In recent years, METH use has remained a significant public health concern worldwide. Publications report that approximately 1.3 million people over the age of 12 in the United States have used METH [106]. Clinical studies have shown that short- and long-term METH abuse affects a wide range of biological processes, including oxidative stress, apoptosis in dopaminergic cell lines, endoplasmic reticulum stress, and microtubule deacetylation [107]. Dang et al. treated cerebral organoids with METH to investigate the effects of drugs on fetal brain development [32]. Differential gene expression analysis showed that METH treatment resulted in the upregulated expression of genes related to inflammation/immune and oxidative stress responses and the downregulated expression of genes involved in neurogenesis and development in cerebral organoids. Additionally, activated astrocytes and a high content of factors related to inflammation and immunity, such as IL-6 and NLRP1, were detected in METH-treated organoids. These results proved that METH treatment could induce neuroinflammation in brain organoids and showed how effectively brain organoids represent a model system for studying complex neuroinflammatory diseases [108]. The appearance of the immune environment in brain organoids will further expand their application in the study of neuroinflammation and immunity.

### Screening antitumor drugs

In recent years, a variety of antitumor treatments have developed rapidly, such as chemotherapy [44], targeted therapy [109], and immunization therapy [110], having brought positive effects on patient survival. However, different responses to the same treatment can be observed between patients with gliomas in clinical practice, mainly due to the heterogeneity of tumors. The 3D organoids derived from PSCs showed success in modeling neurological disorders, especially GBM, suggesting the potential to be a robust preclinical model for effective antitumor drug screening and to develop personalized treatment strategies for GBM patients.

Shan Bian et al. initiated tumorigenesis by introducing three defined gene aberrations into brain organoids. They treated these three different GBM organoids with the EGFR inhibitor, afatinib, which is currently in a clinical trial for GBM, to examine the potential of GBM organoids in targeted drug testing. After 40 days of treatment, three kinds of GBM organoids exhibited different levels of tumor cell reduction, and the organoid with EGFR overactivation showed the most effective reduction result. This demonstrated that organoids are suitable for the evaluation of drug effects in the context of specific DNA aberrations [50]. Similarly, Jacob et al. treated GBOs, a patient-specific GBM model mentioned above, with the postsurgical standard treatment of GBM (chemotherapy combined with radiotherapy) and targeted therapy [37]. For each treatment, the responses of GBOs derived from different GBMs were heterogeneous, and the effectiveness of the treatments is primarily associated with genetic mutations and pathway enrichment in the patient’s tumor, demonstrating the value of a patient-specific GBM organoid model in developing personalized treatment strategies for GBM patients. Immunotherapy achieved significant success in blood tumors and gradually expanded to solid tumor treatment [111]. Recently, CAR-T cells have been used to target the epidermal growth factor receptor variant III (EGFRvIII) commonly found in GBM [112, 113]. Among GBOs derived from six parental GBMs, CAR-T therapy showed a significant therapeutic effect on GBOs with high percentages of EGFRvIII: the CAR-T cell expansion was increased and EGFRvIII-positive tumor cells were decreased.

The tumor microenvironment plays a vital role in tumor growth and metastasis and helps tumor cells fight against stress and damage from antitumor drugs and radioactive rays [57, 114]. In addition, a lack of a tumor niche remains one of the reasons for the poor predictive value of in vitro drug screening in clinical practice. Linkous et al. performed cytotoxicity assays with brain organoids, aiming to investigate whether a different response to chemotherapeutics exists between 3D cerebral tumor organoids and 2D GSCs [36]. The 827 and 923 glioma stem cells, which are patient-derived glioma stem cells, were used as 2D cultures in the experiments. They cocultured 827 and 923 glioma stem cells with cerebral organoids, generating 827 and 923 GLICOs, respectively, which can provide a 3D tumor microenvironment [36]. Next, they developed cytotoxicity assays and observed cell viability in 2D glioma stem cell culture and 3D GBM models. One week posttreatment, temozolomide (TMZ, 1 mM) reduced cell viability by more than 80% in both 827 and 923 2D GSCs. Similarly, bis-chloroethylnitrosourea (BCNU, 100 mM) reduced cell viability by more than 90% in both GSC lines. Despite this dramatic effect in vitro, TMZ (1 mM) treatment in 827 and 923 GLICOs resulted in only 24% and 43% reductions in tumor growth, respectively. Moreover, BCNU (100 mM) treatment attenuated tumor growth by 91% in 827 GLICOs but only by 5% in 923 GLICOs [36]. This result indicated that isogenic GSC lines are significantly more resistant to the chemotherapeutic drug when grown within the microenvironment provided by the cerebral organoid than when grown under the condition of traditional 2D culture. A report summarized that the overall approval rate of phase I–III clinical trials of preclinical drugs was no more than 13.8%, particularly low for antitumor drugs (3.4%), and that the most common reason for the failure of clinical trials was the lack of efficacy (52%) [115, 116]. Brain organoids may bridge the gap between traditional two-dimensional cell line culture and clinical trials to improve the efficiency and accuracy of drug screening by providing the tumor microenvironment.

## Prospects and challenges

**Fig. 2:**
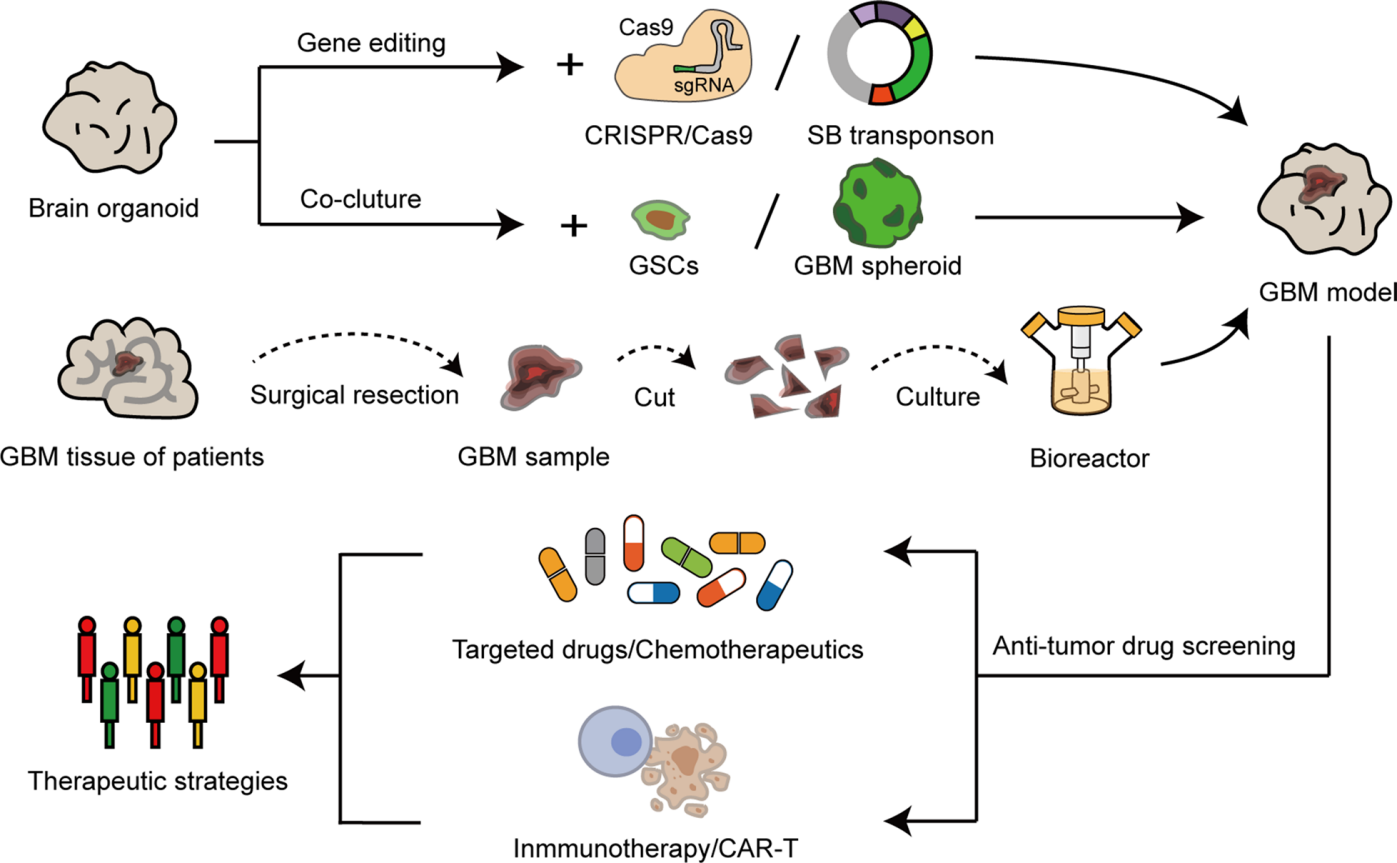
The applications of organoids in GBM modeling and antitumor drug screening. GBM organoid models can be generated by manipulating genes related to tumors with gene editing techniques, or coculturing the glioma stem cells (GSCs) and GBM spheroids derived from human tumors with brain organoids. GBM models can also be constructed by culturing freshly surgically removed tumor samples in optimized medium. In addition, the GBM organoid models have shown tremendous potential in screening effective antitumor drugs and developing the personal treatment for cancer patients

Promising brain organoids have made many achievements in the research of brain development, disease modeling, and regenerative medicine, and have provided a palette for developing personalized therapies, but challenges and limitations still exist (Fig. 2). First, cell diversity in organoids remains to be further enriched. Previous experiments have demonstrated that extended periods of growth and development of organoids promote more cell type generation [33]. Nonetheless, microglial cells, which are derived from hematopoietic stem cells, are still hard to reproduce in brain organoids [117, 118]. Scientists try to coculture the organoids with hemopoietic progenitor cells or combine gene-editing technology to overcome this problem [119]. Second, the lack of blood vessels and the immune environment have been significant obstacles to the use of brain organoids. Recently, vascularized cerebral organoids have been generated, but an immunologic niche remains hard to establish in organoids [28, 29]. It is known that immunization activities maintain a crucial body defense system and interact with almost all kinds of disorders, such as intracranial infection and neurodegenerative diseases [120–122]. Moreover, cancer cells have evolved different mechanisms to simulate peripheral immune tolerance to avoid tumoricidal attack in the process of tumor development [123, 124]. Therefore, overcoming this issue will greatly expand the application scope of brain organoids. Third, a recent study revealed that the brain organoid environment activates cellular stress pathways, which will impair cell-type specification in organoids [38]. In addition, although neuronal populations in organoids include various layer molecular signatures of the cortical plate, they cannot recapitulate a six-layered spatial organization similar to that in human brains [21]. Therefore, the fidelity of organoid models should be considered seriously when probing the developmental process and cell-type-specific diseases of human brains. Last, brain organoids have brought great promise as a cure to patients suffering from neurological disorders, but some significant ethical challenges have arisen, including legal and moral issues [125]. In particular, although brain organoids currently resemble the early embryonic brain, with the development of organoid technology, fine and sophisticated brain organoids may become conscious, such as evoking emotion or developing memories. Hence, the establishment of relevant guidelines and oversight bodies is necessary for the field of brain organoid studies [126].

## Conclusions

As an emerging 3D in vitro model, organoids have played an essential role in promoting medical research and clinical settings. In this review, we provided an overview of the current state of brain organoid differentiation strategies, summarized current applications in the medical domain, and discussed the challenges and prospects. While many points remain to be enhanced, there is no doubt that brain organoids have improved our understanding of the neurodevelopmental process and neurological disorder pathogenesis, and have opened the door to the development of more effective targeted therapies. Increasing emerging technology will inevitably promote the continuous optimization of organoid technology so that it can better serve scientific research.

## Data Availability

Data and materials related to this work are available upon request.

## Abbreviations

2D: Two-dimensional
3D: Three-dimensional
BCNU: Bis-chloroethylnitrosourea
Drop-seq: Droplet-based single-cell mRNA sequencing
DRD1: Dopamine D1 receptor
ESCs: Embryonic stem cells
EBs: Embryoid bodies
ETV2: ETS variant 2
EGFRvIII: Epidermal growth factor receptor variant III
GPCRs: G protein-coupled receptors
GBM: Glioblastoma
GEMMs: Genetically engineered mouse models
GSCs: Glioma stem cells
GLICO: Cerebral organoid glioma
GBO: Glioblastoma organoid
hCOs: Human-cortex-organoids
IFL: Inner fibre layer
iPSCs: Induced pluripotent stem cells
MCPH: Autosomal recessive primary microcephaly
METH: Methamphetamine
NSC: Neural stem cell
NPCs: Neural progenitor cells
OSVZ: Outer subventricular zone
PDXs: Patient-derived xenografts
PSCs: Pluripotent stem cells
RGs: Radial glial stem cells
shCtrl: Control short hairpin RNA
shTP53: Short hairpin RNA against TP53
TP53: Tumor protein p53
TP53KD: TP53-knockdown
TLR3: Toll-like-receptor 3
TMZ: Temozolomide
vhCOs: Vascularized-human-cortex-organoids
ZIKV: Zika virus

## References

[1] Filipkowski RK, Kaczmarek L (2018). Severely impaired adult brain neurogenesis in cyclin D2 knock-out mice produces very limited phenotypic changes. Prog Neuropsychopharmacol Biol Psychiatry.

[2] Comba A, Dunn PJ, Argento AE, Kadiyala P, Ventosa M, Patel P, Zamler DB, Nunez FJ, Zhao L, Castro MG, Lowenstein PR (2020). FYN tyrosine kinase, a downstream target of receptor tyrosine kinases, modulates anti-glioma immune responses. Neuro Oncol.

[3] Mak IW, Evaniew N, Ghert M (2014). Lost in translation: animal models and clinical trials in cancer treatment. Am J Transl Res.

[4] Canals I, Ginisty A, Quist E, Timmerman R, Fritze J, Miskinyte G, Monni E, Hansen MG, Hidalgo I, Bryder D, Bengzon J, Ahlenius H (2018). Rapid and efficient induction of functional astrocytes from human pluripotent stem cells. Nat Methods.

[5] Papapetrou EP (2016). Induced pluripotent stem cells, past and future. Science.

[6] Han F, Liu C, Huang J, Chen J, Wei C, Geng X, Liu Y, Han D, Li M (2019). The application of patient-derived induced pluripotent stem cells for modeling and treatment of Alzheimer’s disease. Brain Sci Adv.

[7] Bryukhovetskiy AS (2018). Translational experience of 28 years of use of the technologies of regenerative medicine to treat complex consequences of the brain and spinal cord trauma: results, problems and conclusions. J Neurorestoratol.

[8] Lancaster MA, Knoblich JA (2014). Organogenesis in a dish: modeling development and disease using organoid technologies. Science.

[9] Lancaster MA, Renner M, Martin CA, Wenzel D, Bicknell LS, Hurles ME, Homfray T, Penninger JM, Jackson AP, Knoblich JA (2013). Cerebral organoids model human brain development and microcephaly. Nature.

[10] Hodge RD, Bakken TE, Miller JA, Smith KA, Barkan ER, Graybuck LT, Close JL, Long B, Johansen N, Penn O, Yao Z, Eggermont J, Hollt T, Levi BP, Shehata SI, Aevermann B, Beller A, Bertagnolli D, Brouner K, Casper T, Cobbs C, Dalley R, Dee N, Ding SL, Ellenbogen RG, Fong O, Garren E, Goldy J, Gwinn RP, Hirschstein D, Keene CD, Keshk M, Ko AL, Lathia K, Mahfouz A, Maltzer Z, McGraw M, Nguyen TN, Nyhus J, Ojemann JG, Oldre A, Parry S, Reynolds S, Rimorin C, Shapovalova NV, Somasundaram S, Szafer A, Thomsen ER, Tieu M, Quon G, Scheuermann RH, Yuste R, Sunkin SM, Lelieveldt B, Feng D, Ng L, Bernard A, Hawrylycz M, Phillips JW, Tasic B, Zeng H, Jones AR, Koch C, Lein ES (2019). Conserved cell types with divergent features in human versus mouse cortex. Nature.

[11] Oberheim NA, Takano T, Han X, He W, Lin JH, Wang F, Xu Q, Wyatt JD, Pilcher W, Ojemann JG, Ransom BR, Goldman SA, Nedergaard M (2009). Uniquely hominid features of adult human astrocytes. J Neurosci.

[12] Boldog E, Bakken TE, Hodge RD, Novotny M, Aevermann BD, Baka J, Borde S, Close JL, Diez-Fuertes F, Ding SL, Farago N, Kocsis AK, Kovacs B, Maltzer Z, McCorrison JM, Miller JA, Molnar G, Olah G, Ozsvar A, Rozsa M, Shehata SI, Smith KA, Sunkin SM, Tran DN, Venepally P, Wall A, Puskas LG, Barzo P, Steemers FJ, Schork NJ, Scheuermann RH, Lasken RS, Lein ES, Tamas G (2018). Transcriptomic and morphophysiological evidence for a specialized human cortical GABAergic cell type. Nat Neurosci.

[13] Wilson HV (1907). A new method by which sponges may be artificially reared. Science.

[14] Thomson JA, Itskovitz-Eldor J, Shapiro SS, Waknitz MA, Swiergiel JJ, Marshall VS, Jones JM (1998). Embryonic stem cell lines derived from human blastocysts. Science.

[15] Martin GR (1981). Isolation of a pluripotent cell line from early mouse embryos cultured in medium conditioned by teratocarcinoma stem cells. Proc Natl Acad Sci USA.

[16] Sato T, Vries RG, Snippert HJ, van de Wetering M, Barker N, Stange DE, van Es JH, Abo A, Kujala P, Peters PJ, Clevers H (2009). Single Lgr5 stem cells build crypt-villus structures in vitro without a mesenchymal niche. Nature.

[17] Dye BR, Hill DR, Ferguson MA, Tsai YH, Nagy MS, Dyal R, Wells JM, Mayhew CN, Nattiv R, Klein OD, White ES, Deutsch GH, Spence JR (2015). In vitro generation of human pluripotent stem cell derived lung organoids. Elife.

[18] Takasato M, Er PX, Becroft M, Vanslambrouck JM, Stanley EG, Elefanty AG, Little MH (2014). Directing human embryonic stem cell differentiation towards a renal lineage generates a self-organizing kidney. Nat Cell Biol.

[19] Chua CW, Shibata M, Lei M, Toivanen R, Barlow LJ, Bergren SK, Badani KK, McKiernan JM, Benson MC, Hibshoosh H, Shen MM. Single luminal epithelial progenitors can generate prostate organoids in culture. Nat Cell Biol. 2014;16(10):951–961, 951–954. 10.1038/ncb3047 PMC4183706 25241035

[20] Chang C, Hemmati-Brivanlou A (1998). Cell fate determination in embryonic ectoderm. J Neurobiol.

[21] Lancaster MA, Knoblich JA (2014). Generation of cerebral organoids from human pluripotent stem cells. Nat Protoc.

[22] Muguruma K, Nishiyama A, Kawakami H, Hashimoto K, Sasai Y (2015). Self-organization of polarized cerebellar tissue in 3D culture of human pluripotent stem cells. Cell Rep.

[23] Qian X, Nguyen HN, Song MM, Hadiono C, Ogden SC, Hammack C, Yao B, Hamersky GR, Jacob F, Zhong C, Yoon KJ, Jeang W, Lin L, Li Y, Thakor J, Berg DA, Zhang C, Kang E, Chickering M, Nauen D, Ho CY, Wen Z, Christian KM, Shi PY, Maher BJ, Wu H, Jin P, Tang H, Song H, Ming GL (2016). Brain-region-specific organoids using mini-bioreactors for modeling ZIKV exposure. Cell.

[24] Jo J, Xiao Y, Sun AX, Cukuroglu E, Tran HD, Goke J, Tan ZY, Saw TY, Tan CP, Lokman H, Lee Y, Kim D, Ko HS, Kim SO, Park JH, Cho NJ, Hyde TM, Kleinman JE, Shin JH, Weinberger DR, Tan EK, Je HS, Ng HH (2016). Midbrain-like organoids from human pluripotent stem cells contain functional dopaminergic and neuromelanin-producing neurons. Cell Stem Cell.

[25] Bagley JA, Reumann D, Bian S, Levi-Strauss J, Knoblich JA (2017). Fused cerebral organoids model interactions between brain regions. Nat Methods.

[26] Xiang Y, Tanaka Y, Patterson B, Kang YJ, Govindaiah G, Roselaar N, Cakir B, Kim KY, Lombroso AP, Hwang SM, Zhong M, Stanley EG, Elefanty AG, Naegele JR, Lee SH, Weissman SM, Park IH (2017). Fusion of regionally specified hPSC-derived organoids models human brain development and interneuron migration. Cell Stem Cell.

[27] Xiang Y, Tanaka Y, Cakir B, Patterson B, Kim KY, Sun P, Kang YJ, Zhong M, Liu X, Patra P, Lee SH, Weissman SM, Park IH (2019). hESC-derived thalamic organoids form reciprocal projections when fused with cortical organoids. Cell Stem Cell.

[28] Mansour AA, Goncalves JT, Bloyd CW, Li H, Fernandes S, Quang D, Johnston S, Parylak SL, Jin X, Gage FH (2018). An in vivo model of functional and vascularized human brain organoids. Nat Biotechnol.

[29] Cakir B, Xiang Y, Tanaka Y, Kural MH, Parent M, Kang YJ, Chapeton K, Patterson B, Yuan Y, He CS, Raredon MSB, Dengelegi J, Kim KY, Sun P, Zhong M, Lee S, Patra P, Hyder F, Niklason LE, Lee SH, Yoon YS, Park IH (2019). Engineering of human brain organoids with a functional vascular-like system. Nat Methods.

[30] Lee S, Park C, Han JW, Kim JY, Cho K, Kim EJ, Kim S, Lee SJ, Oh SY, Tanaka Y, Park IH, An HJ, Shin CM, Sharma S, Yoon YS (2017). Direct reprogramming of human dermal fibroblasts into endothelial cells using ER71/ETV2. Circ Res.

[31] Kanton S, Boyle MJ, He Z, Santel M, Weigert A, Sanchis-Calleja F, Guijarro P, Sidow L, Fleck JS, Han D, Qian Z, Heide M, Huttner WB, Khaitovich P, Paabo S, Treutlein B, Camp JG (2019). Organoid single-cell genomic atlas uncovers human-specific features of brain development. Nature.

[32] Dang J, Tiwari SK, Agrawal K, Hui H, Qin Y, Rana TM (2020). Glial cell diversity and methamphetamine-induced neuroinflammation in human cerebral organoids. Mol Psychiatry.

[33] Quadrato G, Nguyen T, Macosko EZ, Sherwood JL, Min YS, Berger DR, Maria N, Scholvin J, Goldman M, Kinney JP, Boyden ES, Lichtman JW, Williams ZM, McCarroll SA, Arlotta P (2017). Cell diversity and network dynamics in photosensitive human brain organoids. Nature.

[34] Quadrato G, Brown J, Arlotta P (2016). The promises and challenges of human brain organoids as models of neuropsychiatric disease. Nat Med.

[35] Velasco S, Kedaigle AJ, Simmons SK, Nash A, Rocha M, Quadrato G, Paulsen B, Nguyen L, Adiconis X, Regev A, Levin JZ, Arlotta P (2019). Individual brain organoids reproducibly form cell diversity of the human cerebral cortex. Nature.

[36] Linkous A, Balamatsias D, Snuderl M, Edwards L, Miyaguchi K, Milner T, Reich B, Cohen-Gould L, Storaska A, Nakayama Y, Schenkein E, Singhania R, Cirigliano S, Magdeldin T, Lin Y, Nanjangud G, Chadalavada K, Pisapia D, Liston C, Fine HA (2019). Modeling patient-derived glioblastoma with cerebral organoids. Cell Rep.

[37] Jacob F, Salinas RD, Zhang DY, Nguyen PTT, Schnoll JG, Wong SZH, Thokala R, Sheikh S, Saxena D, Prokop S, Liu DA, Qian X, Petrov D, Lucas T, Chen HI, Dorsey JF, Christian KM, Binder ZA, Nasrallah M, Brem S, O'Rourke DM, Ming GL, Song H (2020). A patient-derived glioblastoma organoid model and biobank recapitulates inter- and intra-tumoral heterogeneity. Cell.

[38] Bhaduri A, Andrews MG, Mancia Leon W, Jung D, Shin D, Allen D, Jung D, Schmunk G, Haeussler M, Salma J, Pollen AA, Nowakowski TJ, Kriegstein AR (2020). Cell stress in cortical organoids impairs molecular subtype specification. Nature.

[39] Marin Navarro A, Pronk RJ, van der Geest AT, Oliynyk G, Nordgren A, Arsenian-Henriksson M, Falk A, Wilhelm M (2020). p53 controls genomic stability and temporal differentiation of human neural stem cells and affects neural organization in human brain organoids. Cell Death Dis.

[40] Jaber M, Robinson SW, Missale C, Caron MG (1996). Dopamine receptors and brain function. Neuropharmacology.

[41] Wang Q, Dong X, Lu J, Hu T, Pei G (2020). Constitutive activity of a G protein-coupled receptor, DRD1, contributes to human cerebral organoid formation. Stem Cells.

[42] Li Y, Muffat J, Omer A, Bosch I, Lancaster MA, Sur M, Gehrke L, Knoblich JA, Jaenisch R (2017). Induction of expansion and folding in human cerebral organoids. Cell Stem Cell.

[43] Tan SK, Pastori C, Penas C, Komotar RJ, Ivan ME, Wahlestedt C, Ayad NG (2018). Serum long noncoding RNA HOTAIR as a novel diagnostic and prognostic biomarker in glioblastoma multiforme. Mol Cancer.

[44] Stupp R, Mason WP, van den Bent MJ, Weller M, Fisher B, Taphoorn MJ, Belanger K, Brandes AA, Marosi C, Bogdahn U, Curschmann J, Janzer RC, Ludwin SK, Gorlia T, Allgeier A, Lacombe D, Cairncross JG, Eisenhauer E, Mirimanoff RO, European Organisation for R, Treatment of Cancer Brain T, Radiotherapy G, National Cancer Institute of Canada Clinical Trials G (2005). Radiotherapy plus concomitant and adjuvant temozolomide for glioblastoma. N Engl J Med.

[45] Costa J, Gatermann M, Nimtz M, Kandzia S, Glatzel M, Conradt HS (2018). N-glycosylation of extracellular vesicles from HEK-293 and glioma cell lines. Anal Chem.

[46] Zeng W, Tang Z, Li Y, Yin G, Liu Z, Gao J, Chen Y, Chen F (2020). Patient-derived xenografts of different grade gliomas retain the heterogeneous histological and genetic features of human gliomas. Cancer Cell Int.

[47] Vaubel RA, Tian S, Remonde D, Schroeder MA, Mladek AC, Kitange GJ, Caron A, Kollmeyer TM, Grove R, Peng S, Carlson BL, Ma DJ, Sarkar G, Evers L, Decker PA, Yan H, Dhruv HD, Berens ME, Wang Q, Marin BM, Klee EW, Califano A, LaChance DH, Eckel-Passow JE, Verhaak RG, Sulman EP, Burns TC, Meyer FB, O'Neill BP, Tran NL, Giannini C, Jenkins RB, Parney IF, Sarkaria JN (2020). Genomic and phenotypic characterization of a broad panel of patient-derived xenografts reflects the diversity of glioblastoma. Clin Cancer Res.

[48] Hara T, Verma IM (2019). Modeling gliomas using two recombinases. Cancer Res.

[49] Ogawa J, Pao GM, Shokhirev MN, Verma IM (2018). Glioblastoma model using human cerebral organoids. Cell Rep.

[50] Bian S, Repic M, Guo Z, Kavirayani A, Burkard T, Bagley JA, Krauditsch C, Knoblich JA (2018). Genetically engineered cerebral organoids model brain tumor formation. Nat Methods.

[51] Shergalis A, Bankhead A, Luesakul U, Muangsin N, Neamati N (2018). Current challenges and opportunities in treating glioblastoma. Pharmacol Rev.

[52] Kim EL, Sorokin M, Kantelhardt SR, Kalasauskas D, Sprang B, Fauss J, Ringel F, Garazha A, Albert E, Gaifullin N, Hartmann C, Naumann N, Bikar SE, Giese A, Buzdin A (2020). Intratumoral heterogeneity and longitudinal changes in gene expression predict differential drug sensitivity in newly diagnosed and recurrent glioblastoma. Cancers (Basel).

[53] da Silva B, Mathew RK, Polson ES, Williams J, Wurdak H (2018). Spontaneous glioblastoma spheroid infiltration of early-stage cerebral organoids models brain tumor invasion. SLAS Discov.

[54] Baysan M, Woolard K, Bozdag S, Riddick G, Kotliarova S, Cam MC, Belova GI, Ahn S, Zhang W, Song H, Walling J, Stevenson H, Meltzer P, Fine HA (2014). Micro-environment causes reversible changes in DNA methylation and mRNA expression profiles in patient-derived glioma stem cells. PLoS ONE.

[55] Rubenstein BM, Kaufman LJ (2008). The role of extracellular matrix in glioma invasion: a cellular Potts model approach. Biophys J.

[56] Lu P, Weaver VM, Werb Z (2012). The extracellular matrix: a dynamic niche in cancer progression. J Cell Biol.

[57] Meng X, Duan C, Pang H, Chen Q, Han B, Zha C, Dinislam M, Wu P, Li Z, Zhao S, Wang R, Lin L, Jiang C, Cai J (2019). DNA damage repair alterations modulate M2 polarization of microglia to remodel the tumor microenvironment via the p53-mediated MDK expression in glioma. EBioMedicine.

[58] Osswald M, Jung E, Sahm F, Solecki G, Venkataramani V, Blaes J, Weil S, Horstmann H, Wiestler B, Syed M, Huang L, Ratliff M, Karimian Jazi K, Kurz FT, Schmenger T, Lemke D, Gommel M, Pauli M, Liao Y, Haring P, Pusch S, Herl V, Steinhauser C, Krunic D, Jarahian M, Miletic H, Berghoff AS, Griesbeck O, Kalamakis G, Garaschuk O, Preusser M, Weiss S, Liu H, Heiland S, Platten M, Huber PE, Kuner T, von Deimling A, Wick W, Winkler F (2015). Brain tumour cells interconnect to a functional and resistant network. Nature.

[59] Goranci-Buzhala G, Mariappan A, Gabriel E, Ramani A, Ricci-Vitiani L, Buccarelli M, D'Alessandris QG, Pallini R, Gopalakrishnan J (2020). Rapid and efficient invasion assay of glioblastoma in human brain organoids. Cell Rep.

[60] Fereshtehnejad SM, Zeighami Y, Dagher A, Postuma RB (2017). Clinical criteria for subtyping Parkinson's disease: biomarkers and longitudinal progression. Brain.

[61] Brookmeyer R, Abdalla N, Kawas CH, Corrada MM (2018). Forecasting the prevalence of preclinical and clinical Alzheimer's disease in the United States. Alzheimers Dement.

[62] Talbot K, Feneberg E, Scaber J, Thompson AG, Turner MR (2018). Amyotrophic lateral sclerosis: the complex path to precision medicine. J Neurol.

[63] Gitler AD, Dhillon P, Shorter J (2017). Neurodegenerative disease: models, mechanisms, and a new hope. Dis Model Mech.

[64] Peng C, Trojanowski JQ, Lee VM (2020). Protein transmission in neurodegenerative disease. Nat Rev Neurol.

[65] Liu P, Wu L, Peng G, Han Y, Tang R, Ge J, Zhang L, Jia L, Yue S, Zhou K, Li L, Luo B, Wang B (2019). Altered microbiomes distinguish Alzheimer's disease from amnestic mild cognitive impairment and health in a Chinese cohort. Brain Behav Immun.

[66] Ghosh MC, Zhang DL, Rouault TA (2015). Iron misregulation and neurodegenerative disease in mouse models that lack iron regulatory proteins. Neurobiol Dis.

[67] Keren-Shaul H, Spinrad A, Weiner A, Matcovitch-Natan O, Dvir-Szternfeld R, Ulland TK, David E, Baruch K, Lara-Astaiso D, Toth B, Itzkovitz S, Colonna M, Schwartz M, Amit I (2017). A unique microglia type associated with restricting development of alzheimer's disease. Cell.

[68] Choi H, Kim HJ, Yang J, Chae S, Lee W, Chung S, Kim J, Choi H, Song H, Lee CK, Jun JH, Lee YJ, Lee K, Kim S, Sim HR, Choi YI, Ryu KH, Park JC, Lee D, Han SH, Hwang D, Kyung J, Mook-Jung I (2020). Acetylation changes tau interactome to degrade tau in Alzheimer's disease animal and organoid models. Aging Cell.

[69] Kim H, Park HJ, Choi H, Chang Y, Park H, Shin J, Kim J, Lengner CJ, Lee YK, Kim J (2019). Modeling G2019S-LRRK2 sporadic parkinson's disease in 3D midbrain organoids. Stem Cell Rep.

[70] Osaki T, Uzel SGM, Kamm RD (2018). Microphysiological 3D model of amyotrophic lateral sclerosis (ALS) from human iPS-derived muscle cells and optogenetic motor neurons. Sci Adv.

[71] Karlawish J, Jack CR, Rocca WA, Snyder HM, Carrillo MC (2017). Alzheimer's disease: the next frontier-special report 2017. Alzheimers Dement.

[72] Esquerda-Canals G, Montoliu-Gaya L, Guell-Bosch J, Villegas S (2017). Mouse models of alzheimer's disease. J Alzheimers Dis.

[73] Leng Y, Ackley SF, Glymour MM, Yaffe K, Brenowitz WD (2021). Genetic risk of alzheimer's disease and sleep duration in non-demented elders. Ann Neurol.

[74] Mangialasche F, Solomon A, Winblad B, Mecocci P, Kivipelto M (2010). Alzheimer's disease: clinical trials and drug development. Lancet Neurol.

[75] Nakamura A, Kaneko N, Villemagne VL, Kato T, Doecke J, Dore V, Fowler C, Li QX, Martins R, Rowe C, Tomita T, Matsuzaki K, Ishii K, Ishii K, Arahata Y, Iwamoto S, Ito K, Tanaka K, Masters CL, Yanagisawa K (2018). High performance plasma amyloid-beta biomarkers for Alzheimer's disease. Nature.

[76] Kelleher RJ, Shen J (2017). Presenilin-1 mutations and Alzheimer's disease. Proc Natl Acad Sci USA.

[77] Choi SH, Kim YH, Hebisch M, Sliwinski C, Lee S, D'Avanzo C, Chen H, Hooli B, Asselin C, Muffat J, Klee JB, Zhang C, Wainger BJ, Peitz M, Kovacs DM, Woolf CJ, Wagner SL, Tanzi RE, Kim DY (2014). A three-dimensional human neural cell culture model of Alzheimer's disease. Nature.

[78] Gonzalez C, Armijo E, Bravo-Alegria J, Becerra-Calixto A, Mays CE, Soto C (2018). Modeling amyloid beta and tau pathology in human cerebral organoids. Mol Psychiatry.

[79] Ghatak S, Dolatabadi N, Trudler D, Zhang X, Wu Y, Mohata M, Ambasudhan R, Talantova M, Lipton SA (2019). Mechanisms of hyperexcitability in Alzheimer's disease hiPSC-derived neurons and cerebral organoids vs isogenic controls. Elife.

[80] Dorsey ER, Bloem BR (2018). The parkinson pandemic—a call to action. JAMA Neurol.

[81] Beiske AG, Loge JH, Ronningen A, Svensson E (2009). Pain in Parkinson's disease: prevalence and characteristics. Pain.

[82] Kalia LV, Lang AE (2015). Parkinson's disease. Lancet.

[83] Nijssen J, Comley LH, Hedlund E (2017). Motor neuron vulnerability and resistance in amyotrophic lateral sclerosis. Acta Neuropathol.

[84] Yu CH, Davidson S, Harapas CR, Hilton JB, Mlodzianoski MJ, Laohamonthonkul P, Louis C, Low RRJ, Moecking J, De Nardo D, Balka KR, Calleja DJ, Moghaddas F, Ni E, McLean CA, Samson AL, Tyebji S, Tonkin CJ, Bye CR, Turner BJ, Pepin G, Gantier MP, Rogers KL, McArthur K, Crouch PJ, Masters SL (2020). TDP-43 triggers mitochondrial dna release via mPTP to activate cGAS/STING in ALS. Cell.

[85] Gerbino V, Kaunga E, Ye J, Canzio D, O'Keeffe S, Rudnick ND, Guarnieri P, Lutz CM, Maniatis T (2020). The loss of TBK1 kinase activity in motor neurons or in all cell types differentially impacts ALS disease progression in SOD1 mice. Neuron.

[86] Vinhaes CL, Arriaga MB, de Almeida BL, Oliveira JV, Santos CS, Calcagno JI, Carvalho TX, Giovanetti M, Alcantara LCJ, de Siqueira IC, Andrade BB (2020). Newborns with Zika virus-associated microcephaly exhibit marked systemic inflammatory imbalance. J Infect Dis.

[87] Jayaraman D, Bae BI, Walsh CA (2018). The genetics of primary microcephaly. Annu Rev Genomics Hum Genet.

[88] Bond J, Roberts E, Springell K, Lizarraga SB, Scott S, Higgins J, Hampshire DJ, Morrison EE, Leal GF, Silva EO, Costa SM, Baralle D, Raponi M, Karbani G, Rashid Y, Jafri H, Bennett C, Corry P, Walsh CA, Woods CG (2005). A centrosomal mechanism involving CDK5RAP2 and CENPJ controls brain size. Nat Genet.

[89] Tungadi EA, Ito A, Kiyomitsu T, Goshima G (2017). Human microcephaly ASPM protein is a spindle pole-focusing factor that functions redundantly with CDK5RAP2. J Cell Sci.

[90] Lizarraga SB, Margossian SP, Harris MH, Campagna DR, Han AP, Blevins S, Mudbhary R, Barker JE, Walsh CA, Fleming MD (2010). Cdk5rap2 regulates centrosome function and chromosome segregation in neuronal progenitors. Development.

[91] Gruber R, Zhou Z, Sukchev M, Joerss T, Frappart PO, Wang ZQ (2011). MCPH1 regulates the neuroprogenitor division mode by coupling the centrosomal cycle with mitotic entry through the Chk1-Cdc25 pathway. Nat Cell Biol.

[92] Yingling J, Youn YH, Darling D, Toyo-Oka K, Pramparo T, Hirotsune S, Wynshaw-Boris A (2008). Neuroepithelial stem cell proliferation requires LIS1 for precise spindle orientation and symmetric division. Cell.

[93] Petersen E, Wilson ME, Touch S, McCloskey B, Mwaba P, Bates M, Dar O, Mattes F, Kidd M, Ippolito G, Azhar EI, Zumla A (2016). Rapid spread of Zika virus in the americas-implications for public health preparedness for mass gatherings at the 2016 Brazil olympic games. Int J Infect Dis.

[94] Gladwyn-Ng I, Cordon-Barris L, Alfano C, Creppe C, Couderc T, Morelli G, Thelen N, America M, Bessieres B, Encha-Razavi F, Bonniere M, Suzuki IK, Flamand M, Vanderhaeghen P, Thiry M, Lecuit M, Nguyen L (2018). Stress-induced unfolded protein response contributes to Zika virus-associated microcephaly. Nat Neurosci.

[95] Garcez PP, Loiola EC, Madeiro da Costa R, Higa LM, Trindade P, Delvecchio R, Nascimento JM, Brindeiro R, Tanuri A, Rehen SK (2016). Zika virus impairs growth in human neurospheres and brain organoids. Science.

[96] Khongwichit S, Sornjai W, Jitobaom K, Greenwood M, Greenwood MP, Hitakarun A, Wikan N, Murphy D, Smith DR (2021). A functional interaction between GRP78 and Zika virus E protein. Sci Rep.

[97] Laguesse S, Creppe C, Nedialkova DD, Prevot PP, Borgs L, Huysseune S, Franco B, Duysens G, Krusy N, Lee G, Thelen N, Thiry M, Close P, Chariot A, Malgrange B, Leidel SA, Godin JD, Nguyen L (2015). A dynamic unfolded protein response contributes to the control of cortical neurogenesis. Dev Cell.

[98] Tseng KY, Danilova T, Domanskyi A, Saarma M, Lindahl M, Airavaara M (2017). MANF is essential for neurite extension and neuronal migration in the developing cortex. eNeuro.

[99] Tang H, Hammack C, Ogden Sarah C, Wen Z, Qian X, Li Y, Yao B, Shin J, Zhang F, Lee Emily M, Christian Kimberly M, Didier Ruth A, Jin P, Song H, Ming G-L (2016). Zika virus infects human cortical neural progenitors and attenuates their growth. Cell Stem Cell.

[100] Dang J, Tiwari SK, Lichinchi G, Qin Y, Patil VS, Eroshkin AM, Rana TM (2016). Zika virus depletes neural progenitors in human cerebral organoids through activation of the innate immune receptor TLR3. Cell Stem Cell.

[101] Setoh YX, Amarilla AA, Peng NYG, Griffiths RE, Carrera J, Freney ME, Nakayama E, Ogawa S, Watterson D, Modhiran N, Nanyonga FE, Torres FJ, Slonchak A, Periasamy P, Prow NA, Tang B, Harrison J, Hobson-Peters J, Cuddihy T, Cooper-White J, Hall RA, Young PR, Mackenzie JM, Wolvetang E, Bloom JD, Suhrbier A, Khromykh AA (2019). Determinants of Zika virus host tropism uncovered by deep mutational scanning. Nat Microbiol.

[102] Driggers RW, Ho CY, Korhonen EM, Kuivanen S, Jaaskelainen AJ, Smura T, Rosenberg A, Hill DA, DeBiasi RL, Vezina G, Timofeev J, Rodriguez FJ, Levanov L, Razak J, Iyengar P, Hennenfent A, Kennedy R, Lanciotti R, du Plessis A, Vapalahti O (2016). Zika virus infection with prolonged maternal viremia and fetal brain abnormalities. N Engl J Med.

[103] De Miranda J, Yaddanapudi K, Hornig M, Villar G, Serge R, Lipkin WI, Rall G, Biron CA. Induction of Toll-like receptor 3-mediated immunity during gestation inhibits cortical neurogenesis and causes behavioral disturbances. mBio 2010;1(4). 10.1128/mBio.00176-10 PMC2953007 20941330

[104] Okun E, Griffioen K, Barak B, Roberts NJ, Castro K, Pita MA, Cheng A, Mughal MR, Wan R, Ashery U, Mattson MP (2010). Toll-like receptor 3 inhibits memory retention and constrains adult hippocampal neurogenesis. Proc Natl Acad Sci.

[105] Weafer J, Van Hedger K, Keedy SK, Nwaokolo N, de Wit H (2020). Methamphetamine acutely alters frontostriatal resting state functional connectivity in healthy young adults. Addict Biol.

[106] Prakash MD, Tangalakis K, Antonipillai J, Stojanovska L, Nurgali K, Apostolopoulos V (2017). Methamphetamine: effects on the brain, gut and immune system. Pharmacol Res.

[107] Chang L, Alicata D, Ernst T, Volkow N (2007). Structural and metabolic brain changes in the striatum associated with methamphetamine abuse. Addiction.

[108] Du S-H, Qiao D-F, Chen C-X, Chen S, Liu C, Lin Z, Wang H, Xie W-B (2017). Toll-like receptor 4 mediates methamphetamine-induced neuroinflammation through caspase-11 signaling pathway in astrocytes. Front Mol Neurosci.

[109] Lin L, Cai J, Jiang C (2017). Recent advances in targeted therapy for glioma. Curr Med Chem.

[110] Schijns V, Pretto C, Strik AM, Gloudemans-Rijkers R, Deviller L, Pierre D, Chung J, Dandekar M, Carrillo JA, Kong XT, Fu BD, Hsu FPK, Hofman FM, Chen TC, Zidovetzki R, Bota DA, Stathopoulos A (2018). Therapeutic immunization against glioblastoma. Int J Mol Sci.

[111] Feins S, Kong W, Williams EF, Milone MC, Fraietta JA (2019). An introduction to chimeric antigen receptor (CAR) T-cell immunotherapy for human cancer. Am J Hematol.

[112] Choi BD, Yu X, Castano AP, Bouffard AA, Schmidts A, Larson RC, Bailey SR, Boroughs AC, Frigault MJ, Leick MB, Scarfo I, Cetrulo CL, Demehri S, Nahed BV, Cahill DP, Wakimoto H, Curry WT, Carter BS, Maus MV (2019). CAR-T cells secreting BiTEs circumvent antigen escape without detectable toxicity. Nat Biotechnol.

[113] Yang J, Yan J, Liu B (2017). Targeting EGFRvIII for glioblastoma multiforme. Cancer Lett.

[114] Chen Q, Han B, Meng X, Duan C, Yang C, Wu Z, Magafurov D, Zhao S, Safin S, Jiang C, Cai J (2019). Immunogenomic analysis reveals LGALS1 contributes to the immune heterogeneity and immunosuppression in glioma. Int J Cancer.

[115] Harrison RK (2016). Phase II and phase III failures: 2013–2015. Nat Rev Drug Discov.

[116] Wong CH, Siah KW, Lo AW (2019). Estimation of clinical trial success rates and related parameters. Biostatistics.

[117] Stratoulias V, Venero JL, Tremblay ME, Joseph B (2019). Microglial subtypes: diversity within the microglial community. EMBO J.

[118] Song L, Yan Y, Marzano M, Li Y (2019). Studying heterotypic cell(-)cell interactions in the human brain using pluripotent stem cell models for neurodegeneration. Cells.

[119] Abud EM, Ramirez RN, Martinez ES, Healy LM, Nguyen CHH, Newman SA, Yeromin AV, Scarfone VM, Marsh SE, Fimbres C, Caraway CA, Fote GM, Madany AM, Agrawal A, Kayed R, Gylys KH, Cahalan MD, Cummings BJ, Antel JP, Mortazavi A, Carson MJ, Poon WW, Blurton-Jones M (2017). iPSC-derived human microglia-like cells to study neurological diseases. Neuron.

[120] Sato R, Kato A, Chimura T, Saitoh SI, Shibata T, Murakami Y, Fukui R, Liu K, Zhang Y, Arii J, Sun-Wada GH, Wada Y, Ikenoue T, Barber GN, Manabe T, Kawaguchi Y, Miyake K (2018). Combating herpesvirus encephalitis by potentiating a TLR3-mTORC2 axis. Nat Immunol.

[121] Bianchi E, Roncarati P, Hougrand O, Guerin-El Khourouj V, Boreux R, Kroonen J, Martin D, Robe P, Rogister B, Delvenne P, Deprez M (2015). Human cytomegalovirus and primary intracranial tumours: frequency of tumour infection and lack of correlation with systemic immune anti-viral responses. Neuropathol Appl Neurobiol.

[122] Yazdani S, Mariosa D, Hammar N, Andersson J, Ingre C, Walldius G, Fang F (2019). Peripheral immune biomarkers and neurodegenerative diseases: a prospective cohort study with 20 years of follow-up. Ann Neurol.

[123] Barnet MB, Blinman P, Cooper W, Boyer MJ, Kao S, Goodnow CC (2018). Understanding immune tolerance of cancer: re-purposing insights from fetal allografts and microbes. BioEssays.

[124] Marigo I, Bosio E, Solito S, Mesa C, Fernandez A, Dolcetti L, Ugel S, Sonda N, Bicciato S, Falisi E, Calabrese F, Basso G, Zanovello P, Cozzi E, Mandruzzato S, Bronte V (2010). Tumor-induced tolerance and immune suppression depend on the C/EBPbeta transcription factor. Immunity.

[125] Sawai T, Sakaguchi H, Thomas E, Takahashi J, Fujita M (2019). The ethics of cerebral organoid research: being conscious of consciousness. Stem Cell Rep.

[126] Chen HI, Wolf JA, Blue R, Song MM, Moreno JD, Ming GL, Song H (2019). Transplantation of human brain organoids: revisiting the science and ethics of brain chimeras. Cell Stem Cell.

